# Nitrogen uptake dynamics of high and low protein wheat genotypes

**DOI:** 10.3389/fpls.2024.1493901

**Published:** 2024-12-16

**Authors:** Samson Olaniyi Abiola, Josefina Lacasa, Brett F. Carver, Brian D. Arnall, Ignacio A. Ciampitti, Amanda de Oliveira Silva

**Affiliations:** ^1^ Department of Plant and Soil Sciences, Oklahoma State University, Stillwater, OK, United States; ^2^ Department of Statistics, Kansas State University, Manhattan, KS, United States; ^3^ Department of Agronomy, Kansas State University, Manhattan, KS, United States

**Keywords:** nitrogen use efficiency, nutrient partitioning, crop physiology, genotype selection, plant nutrition, breeding strategies, agronomic practices, N remobilization patterns

## Abstract

Increasing wheat (*Triticum aestivum* L.) yield and grain protein concentration (GPC) without excessive nitrogen (N) inputs requires understanding the genotypic variations in N accumulation, partitioning, and utilization strategies. This study evaluated whether high protein genotypes exhibit increased N accumulation (herein also expressed as N nutrition index, NNI) and partitioning (including remobilization from vegetative organs) compared to low-protein genotypes under low and high N conditions. Four winter wheat genotypes with similar yields but contrasting GPC were examined under two N rates (0 and 120 kg N ha^-1^) across two environments and four growing seasons in Oklahoma, US. As expected, the high-protein genotypes Doublestop CL+ (Dob) and Green Hammer (Grn) had greater GPC than the medium- (Gallagher, Gal) and low-protein genotypes (Iba), without any difference in grain yield. Total plant N accumulation at maturity showed diminishing increases for greater grain yield, and low-protein genotype showed greater N utilization efficiency (NUtE) than high-protein genotypes. The high-protein genotype Grn tended to achieve higher GPC by increasing total N uptake, while Dob exhibited a tendency towards higher N partitioning to grain (NHI). The allometric relationship between total N accumulation and biomass remained unchanged for both high- and low-protein genotypes. The N remobilization patterns differed between high- and low-protein genotypes. As N conditions improved, the proportional contributions of remobilized N from leaves tended to increase, while contributions from stems and chaff tended to decrease or remained unchanged for high-protein genotypes. This study highlights the importance of both N uptake capacity and efficient N partitioning to the grain as critical traits for realizing wheat’s dual goals of higher yield and protein. Leaf N remobilization plays a critical role during grain filling, sustaining plant N status and contributing to protein levels. The higher NUtE observed in the low-protein genotype Iba likely contributed to its lower GPC, emphasizing the trade-off between NUtE and GPC. The physiological strategies employed by high-protein genotypes, such as genotype Grn’s tendency for increased N uptake and Dob’s efficient N partitioning, provide a foundation for future breeding efforts aimed at developing resource-efficient and nutritionally superior wheat genotypes capable of achieving both increased yield and protein.

## Introduction

Wheat (*Triticum aestivum* L.) is the most widely cultivated staple cereal crop, providing calories to 20% of the world’s population and serving as a vital source of human nutrition (FAO, 2019; Padhy et al., 2024). Given its growing global demand and importance for food security, improving grain yield and grain protein concentration (GPC) is crucial to meet the rising production needs and the nutritional quality of wheat (Sendhil et al., 2022; Teng et al., 2022). However, simultaneously enhancing grain yield and GPC is challenging due to their trade-off (Michel et al., 2019). This trade-off occurs because increasing both grain yield (a C-based compound) and GPC (an N compound) are competing metabolic processes for energy (Munier-Jolain and Salon, 2005). Furthermore, the dilution theory suggests that as grain yield increases, protein concentration decreases due to the dilution of N in the grain (i.e., source limitation) (Whitfield and Smith, 1992; Acreche and Slafer, 2009). However, this negative relationship between yield and GPC can be mitigated or even reversed through improved crop management practices and genotype selection (Zhang et al., 2017; Pan et al., 2020). It has been postulated that selecting genotypes with high total N uptake at maturity, N harvest index (NHI, ratio of grain N to total N uptake), N accumulation during grain filling (PostN) or N remobilization from vegetative organs to the grain (RemN) are associated with ability of genotypes to reach adequate yield while maintaining or improving its GPC (Bogard et al., 2010; Cormier et al., 2016; Fortunato et al., 2023). However, earlier research has reported inconsistent associations between NHI, PostN, RemN, and GPC under different environmental conditions. For instance, in wheat genotypes with different protein levels cultivated under dryland conditions, Desai and Bhatia (1978) and Chen et al. (2023) found a weak relationship between NHI and GPC. In general, RemN has contributed from 60 to 95% to grain N at maturity as compared to PostN, which has provided a 35 to 55% contribution to grain N accumulation (Palta and Fillery, 1995; Kichey et al., 2007). However, these contributions may differ depending on the environment, genotype, and management techniques (Taulemesse et al., 2015). Given these inconsistencies, evaluating the physiological mechanisms responsible for reducing the yield-protein trade-off is very important. Understanding these mechanisms may provide insights into the drivers of genotypic variability in GPC and help breeding programs identify wheat genotypes that can achieve high yield and GPC.

Although the literature is vast in evaluating the contribution of RemN to GPC (Barbottin et al., 2005; Barraclough et al., 2010; Gaju et al., 2014; Chen et al., 2015; Kaur et al., 2015), it lacks an understanding of the relative contributions of N remobilization from different vegetative organs (e.g., leaves, stems, chaff) to the grain in modern genotypes. Moreover, the negative relationship between N utilization efficiency (ratio of grain yield and total N uptake, NUtE) and GPC highlights the difficulty of breeding and managing crops to maximize grain yield and GPC concurrently (Gaju et al., 2014; Thompson et al., 2022). N utilization efficiency measures the capacity of genotypes to make use of the N accumulated by the plant for grain or biomass production (Moll et al., 1982; De Oliveira Silva et al., 2020; Lebedev et al., 2021; Biradar et al., 2024). Thus, examining the dynamics of NUtE may offer important insights into the physiological mechanisms controlling the yield-protein trade-off.

The Nitrogen Nutrition Index (NNI), defined as the ratio between the actual plant N concentration and the critical N concentration required for maximum growth, serves as a valuable indicator of crop N status throughout the growing season (Lemaire et al., 2008; Sadras and Lemaire, 2014). Thus, evaluating the association of physiological traits and NNI (e.g., NNI and yield components, Rodriguez et al., 2024), could provide crucial insights into the plant’s nitrogen nutritional state, allowing for a more nuanced understanding of nitrogen dynamics in relation to yield and protein formation.

Our study aims to provide a comprehensive understanding of the physiological mechanisms underlying genotypic differences in GPC under high and low N conditions in dryland environments to ultimately inform breeding strategies and crop management practices to develop genotypes capable of optimizing both yield and protein concentration. Specifically, our objectives were to (a) evaluate whether high-protein genotypes exhibit increased N accumulation and partitioning as compared to medium-low protein genotypes under high and low N availability and (b) investigate whether the relationship between the proportional contributions of N remobilization from each vegetative organ to the grain and NNI differs between high and low protein genotypes.

## Material and methods

### General experiment information

A non-irrigated research study was conducted in two locations for four years (i.e., eight site-years) in Oklahoma. The experiments were established at the Oklahoma State University Research Station in Stillwater (36°08’24.9’N, 9705’37.0”W) and the Cimarron Valley Research Station in Perkins (35°59’25.2”N, 97°02’41.2”W) during 2019-2020, 2020-2021, 2021-2022 and 2022-2023 wheat growing seasons. The soil type for the experiment in Stillwater was Port silt loam (Fine-silty, mixed, superactive, thermic Cumulic Haplustolls) (USDA/NRCS soil taxonomy). The soil type for the experiment carried out in Perkins was the Teller series (fine loamy, mixed, active, thermic Udic Argiustolls) and Konawa series (fine loamy, mixed, active, thermic Ultic Haplustalfs). Weather information was acquired daily from planting to harvest (i.e., from early October to the end of June) from automated weather stations operated by the Mesonet Oklahoma weather network, proximately located to the research sites (**Table 1**).

**Table 1:** Weather information.

Year	Site	Season^s^	Cum PPT	10-yr Cum PPT	T max	T min	T avg	10-yr T max	10-yr T min	10-yr T avg	Cum GDD
			——— mm ———		——————————————–°C—————————————						
2019-2020	Stillwater	Fall	134	177	33	-11	8	27	-8	6	364
		Winter	219	122	34	-11	7	26	-12	19	1004
		Spring	141	331	36	-1	19	34	5	26	2814
	Perkins	Fall	185	153	33	-10	9	27	-6	6	439
		Winter	267	118	33	-9	8	25	-10	21	1170
		Spring	177	382	37	-1	20	34	6	26	2784
2020-2021	Stillwater	Fall	220	198	18	3	10	22	-2	8	586
		Winter	137	129	12	-2	5	19	-7	19	1256
		Spring	108	219	22	9	19	28	7	23	2560
	Perkins	Fall	223	204	17	4	10	25	-1	10	582
		Winter	133	126	11	-1	5	22	-10	7	1218
		Spring	243	313	25	14	19	31	6	20	2363
2021-2022	Stillwater	Fall	127	163	20	6	13	21	2	10	829
		Winter	117	123	20	10	5	19	2	9	1518
		Spring	421	320	39	15	21	34	11	22	2666
	Perkins	Fall	144	174	20	5	12	23	2	11	908
		Winter	100	113	13	-3	5	18	-7	6	1609
		Spring	409	360	28	15	21	29	10	21	2584
2022-2023	Stillwater	Fall	160	179	19	6	9	23	160	11	364
		Winter	157	123	20	11	5	19	157	9	1013
		Spring	223	314	39	16	22	32	223	21	2822
	Perkins	Fall	154	169	20	8	12	22	154	12	441
		Winter	100	105	13	-1	5	17	100	6	1180
		Spring	405	354	26	13	21	29	405	22	2787

Fall: October to December; Winter: January to March; Spring: April to June.

Cumulative precipitation (Cum PPT) in millimeters, maximum, minimum, and average daily temperature (T) in Celsius for each site-year and season, and average of 10 years (2013-2023), cumulative growing degree-day (Cum GDD) in Celsius for each site-year and season.

The field trials in Perkins were performed under conventional tillage, implemented in the fall before wheat sowing, and under a no-tillage system in Stillwater. The experiments were sown using a Great Plains Not-till drill (3P605NT) with a seeding rate of two million seeds ha^-1^. Plots were 10.6 m long and 1.5 m wide, with seven rows 0.19 m apart. At sowing, 47 kg ha^-1^ of ammonium polyphosphate (10-34-0) was applied along the seed furrow in all plots. Diseases, insects, and weeds were chemically controlled as needed. Soil fertility was evaluated at the time of sowing and after harvesting for each site-year (**Table 2**). At sowing, 15 soil cores were obtained at 0-6 inches and 6-12 inches depth across the field and combined to represent a composite sample using hand probes. Two weeks after harvest, soil samples were collected from each individual plot at the same depths (0-15 cm and 15-30 cm) using hand probes. Samples were analyzed at the Oklahoma State University’s Soil, Water, and Forage Analytical Laboratory (SWFAL) to analyze pH, N, P, K, Mg, Ca, and SO_4_ (**Table 2**).

**Table 2:** Soil fertility information at sowing and after harvest for each site-year.

Soil sampling timing	Growing season	Location	pH	N-Surface	N-Subsoil	P	K	SO_4_	Ca	Mg
				——–kg ha^-1^ ———		— ppm —–		—————kg ha^-1^————–		
At sowing	2019-2020	Stillwater	5.5	11	54	19	159			
		Perkins	6.4	11	7	102	255	10	1806	453
	2020-2021	Stillwater	5.3	21	36	56	83	13	1837	560
		Perkins	6.1	12	5	104	260	12	1215	313
	2021-2022	Stillwater	5.4	43	8	54	172			
		Perkins	6.2	26	8	106	237			
	2022-2023	Stillwater	5.1	14	16	56	200	2	1580	489
		Perkins	5.6	25	61	112	264	1	1151	489
After harvest	2019-2020	Stillwater	5.5	11	5	19	158			
		Perkins	6.3	11	9	68	209			
	2020-2021	Stillwater	5.4	21	14	42	146			
		Perkins	6.1	12	11	76	199			
	2022-2023	Stillwater	5.6	14	7	45	146			
		Perkins	6.1	25	6	67	177			

Empty spaces; data not available.

The variables measured include soil pH, total N in the soil surface (0-15 cm) and sub-soil (15-30 cm), Mehlich-3 extractable phosphorus (P), potassium (K), sulfur (SO_4_), calcium (Ca), and magnesium (Mg).

### Experimental design and treatment structure

The experiment was arranged in a randomized complete block design (RCBD) with a 2x4 factorial treatment structure (i.e., two N rates and four genotypes) and four replications. The N rates evaluated were zero N (0N) application, which represented the conditions with only the residual soil N pool, and 120 kg N ha^-1^ (120N) of urea (46-0-0) broadcasted in the fall, which represented conditions with an appropriate fertilizer application for the region (Zhang et al., 2017). The four winter wheat genotypes evaluated were Iba with Plant Variety Protection Act (PVP, 201300135), released in 2012; Gallagher (Gal) (PI 667569; PV 201300134), released in 2012; Doublestop CL+ (Dob) (PVP 201400228), released in 2013; and Green Hammer (Grn) (PVP 201900171), released in 2018. These genotypes were selected due to their adaptability to the environments explored in this study, their similar yield levels, and differences in grain protein concentration (GPC). Iba represented a low protein genotype, Gal a medium protein genotype, and Dob and Grn high protein genotypes (De Oliveira Silva et al., 2023).

### Aboveground plant biomass harvesting, partitioning, and total N analysis

Aboveground biomass was collected from one linear meter of a middle row at anthesis (Z59) and physiological maturity (Z92) at four replications. In the first and second growing seasons (i.e., 2019-2020 and 2020-2021), aboveground biomass was collected by the average growth stage of all genotypes. In the following two growing seasons (i.e., 2021-2022 and 2022-2023), samples were collected based on the growth stage of each genotype. Plants were cut with an electric clipper as close to the soil surface as possible at each growth stage. Plants were partitioned into leaf, stem, and spike at anthesis and leaf, stem, grain, and chaff at physiological maturity. Then, samples were placed in a dryer set at 65°C for seven days. The dry weight of each plant organ was recorded, and plant samples were sent to the OSU Soil, Water and Forage Analytical Laboratory for total N analysis using a LECO CN Combustion Analyzer and an Elementa CN Combustion Analyzer. Grain yield was estimated from the aboveground biomass samples collected at physiological maturity.

Plant N uptake was calculated by multiplying the aboveground biomass of each plant organ by its N concentration (%) (Ciampitti and Vyn, 2013), as


(1) $$\begin{array}{c} \text{Plant N uptake }\left({\text{kg ha}}^{-1}\right)\\ =\text{Plant biomass organ }\left({\text{kg ha}}^{-1}\right)\times \text{ \% N organ } \end{array}$$

Grain protein concentration (GPC) was calculated by multiplying the grain N concentration (%) by the conversion factor 5.7 (i.e., 5.7 units of protein per unit of N) (Sosulski and Imafidon, 1990), as


(2) GPC (%)= Grain N concentration (%)×5.7

The Nitrogen Harvest Index (NHI, %) was estimated as the ratio of grain N uptake to whole plant N uptake at physiological maturity (Ciampitti and Vyn, 2012), as


(3) $$\text{NHI }\left(\%\right)=\frac{\text{Grain N uptake}}{\text{Total N Uptake }}\times 100$$

Post-flowering N uptake (PostN, kg ha^-1^) was calculated as the whole plant N uptake at physiological maturity minus whole plant N uptake at anthesis, as


(4) $$\begin{array}{c} \text{PostN }\left({\text{kg ha}}^{-1}\right)\\ =\text{ whole plant N uptake at maturity}\\ -\text{whole plant N uptake at anthesis} \end{array}$$

Remobilized N (RemN, kg ha^-1^) was calculated as whole plant N uptake at the anthesis stage minus stover N uptake (i.e., stem + leaf + chaff) at the physiological maturity stage, as


(5) $$\begin{array}{c} \text{RemN }\left({\text{kg ha}}^{-1}\right)\\ =\text{ whole plant N uptake at anthesis}\\ -\\ \text{stover N uptake at maturity} \end{array}$$

Nitrogen remobilized from individual vegetative organs (organ Rem N, kg ha^-1^) was calculated as the difference between the N uptake of each vegetative organ (i.e., stem, leaf, and spike) at anthesis and N uptake in the same organs at maturity (Ortez et al., 2019), as


(6) $${\text{RemN}}_{\text{Organ }}\left({\text{kg ha}}^{-1}\right)=\;\text{N uptake organ at anthesis}-\text{N uptake organ at physiological maturity}$$

Nitrogen remobilized from all vegetative organs from anthesis to maturity (Sum RemN_Veg_) was calculated as the sum of the remobilized N in the three vegetative plant organs (i.e., stem, leaf, and chaff) (Ortez et al., 2019), as


(7) $${\text{Sum RemN}}_{\text{Veg}}\left({\text{kg ha}}^{-1}\right)={\text{RemN}}_{\text{leaf}}+{\text{RemN}}_{\text{stem}}+{\text{RemN}}_{\text{chaff}}$$

The contribution of N remobilization from each vegetative plant organ ( ${\text{Contribution RemN}}_{\text{organ}},\;$ i.e., stem, leaf, or chaff) was estimated as relative to the total vegetative N remobilization (Ortez et al., 2019), as


(8) $$\text{Contribution RemN organ }\left(\%\right)=\frac{{\text{RemN}}_{\text{Organ }}}{{\text{Sum RemN}}_{\text{Veg}}}\times 100$$

The actual plant N concentration (N_a_, %) was estimated by dividing the whole plant N uptake (N_a_, %) by whole plant biomass at anthesis, as


(9) $${\text{N}}_{a}\left(\%\right)=\frac{\text{Whole plant N uptake at Anthesis}}{\text{Whole plant biomass at Anthesis}}\times 100$$

Critical N concentration (N_c_, %) was determined using the critical dilution curve model for wheat from Justes et al. (1994), where 5.35% was the whole plant N concentration, when biomass was between 1.5 to 12 Mg ha^-1^ and -0.442 the dilution coefficient (i.e., rate of decrease in whole plant N concentration as the biomass increases), as


(10) $${\text{N}}_{c}\left(\%\right)\;=5.35\times {\text{biomass}}^{-0.422\;}\;$$

The Nitrogen Nutrition Index (NNI) (Equation 11) was estimated by dividing the actual N concentration of whole plant (N_a_, %) at anthesis (Equation 9) by the critical N concentration (N_c_, %) (Equation 10), as


(11) $$\text{NNI }\left(\text{dimmensionless}\right)=\;\frac{N_{a}}{N_{c}}$$

### Statistical analysis

Linear mixed models were applied for evaluating the treatment effect on the traits measured. For each trait at each location, we fitted the model i.e., we considered genotype, N rate, and their interactions as fixed effects, and blocks nested within each year as random effects. The models can be generally described as


(12) $$y_{ijkl}=\mu +\tau_{i}+\rho_{j}+{\left(\tau \rho \right)}_{ij}+u_{k}+v_{kl}+\epsilon_{ijkl},$$

where y_ijkl_ is the trait being studied observed for the ith genotype, jth N treatment, kth year and lth block, µ is the overall mean, $\tau_{i}$ is the effect of the *i*th genotype, $\rho_{j}$ is the effect of the *j*th N treatment, ${\left(\tau \rho \right)}_{ij}$ is the interaction between the *i*th genotype and *j*th N treatment, $u_{k}∼N\left(0,\;\sigma_{u}^{2}\right)$ is the random effect of the kth year, $v_{kl}∼N\left(0,\;\sigma_{v}^{2}\right)$ is the random effect of the *l*th block in the *k*th year, and ϵ is the residual. All three *u*, *v*, and ϵ are assumed iid and independent among each other.

For the remobilized N and its contribution to grain N accumulation, we fit the model


(13) $$\begin{array}{c} y_{ijkl}=\mu +\tau_{i}+\rho_{j}+\gamma_{k}+{\left(\tau \rho \right)}_{ij}+{\left(\tau \gamma \right)}_{ik}+{\left(\rho \gamma \right)}_{jk}+{\left(\tau \rho \gamma \right)}_{ijk}+u_{l}\\ +v_{lm}+\epsilon_{ijklm}, \end{array}$$

where *y_ijklm_* is the trait being studied observed for the *i*th genotype, *j*th N treatment, *k*th year, *l*th block at the *m*th location, µ is the overall mean, $\tau_{i}$ is the effect of the *i*th genotype, $\rho_{j}$ is the effect of the *j*th N treatment, ${\left(\tau \rho \right)}_{ij}$ , ${\left(\tau \gamma \right)}_{ik}$ , ${\left(\rho \gamma \right)}_{jk}$ are the two-way interactions, ${\left(\tau \rho \gamma \right)}_{ijk}$ is the three way interaction, is the random effect of the *l*th year, $v_{lm}∼N\left(0,\;\sigma_{v}^{2}\right)$ is the random effect of the *m*th block in the *l*th year, and ϵ is the residual. All three *u*, *v*, and ϵ are assumed iid and independent among each other. We performed an ANOVA using the models outlined in Equation 12 for the major traits (i.e., yield, GPC, plant biomass, plant N uptake). Next, we compared mean differences between treatments (i.e., combinations of N treatment and genotype) within locations using the Tukey adjustments and a significance level of 0.05.

To describe the relationship between grain yield *vs* plant N uptake at maturity, total biomass *vs* N uptake at maturity, and grain N accumulation *vs* N uptake at maturity, we compared simple linear regression (i.e., intercept and slope) to quadratic, and cubic. All models were fitted using R software. Linear mixed models were fitted using the R packages “lme4” (Bates et al., 2015) and “lmerTest” (Kuznetsova et al., 2017), and multiple comparisons were done with the “multcomp” package in R (Hothorn et al., 2008).

## Results

### Examining grain yield, grain protein concentration, and NUtE trends in winter wheat genotypes

This study evaluated the ability of genotypes to produce grain yield while minimizing the trade-off with GPC by evaluating four winter wheat genotypes (selected for their known similar yield levels and varying GPC), two N rates (0N and 120N), two locations, and four growing seasons (from 2019-2020 to 2022-2023) resulting in a total of 256 observations. The results were reported for each site across years (i.e., individual sites were analyzed separately) independently.

**Figure 1:**
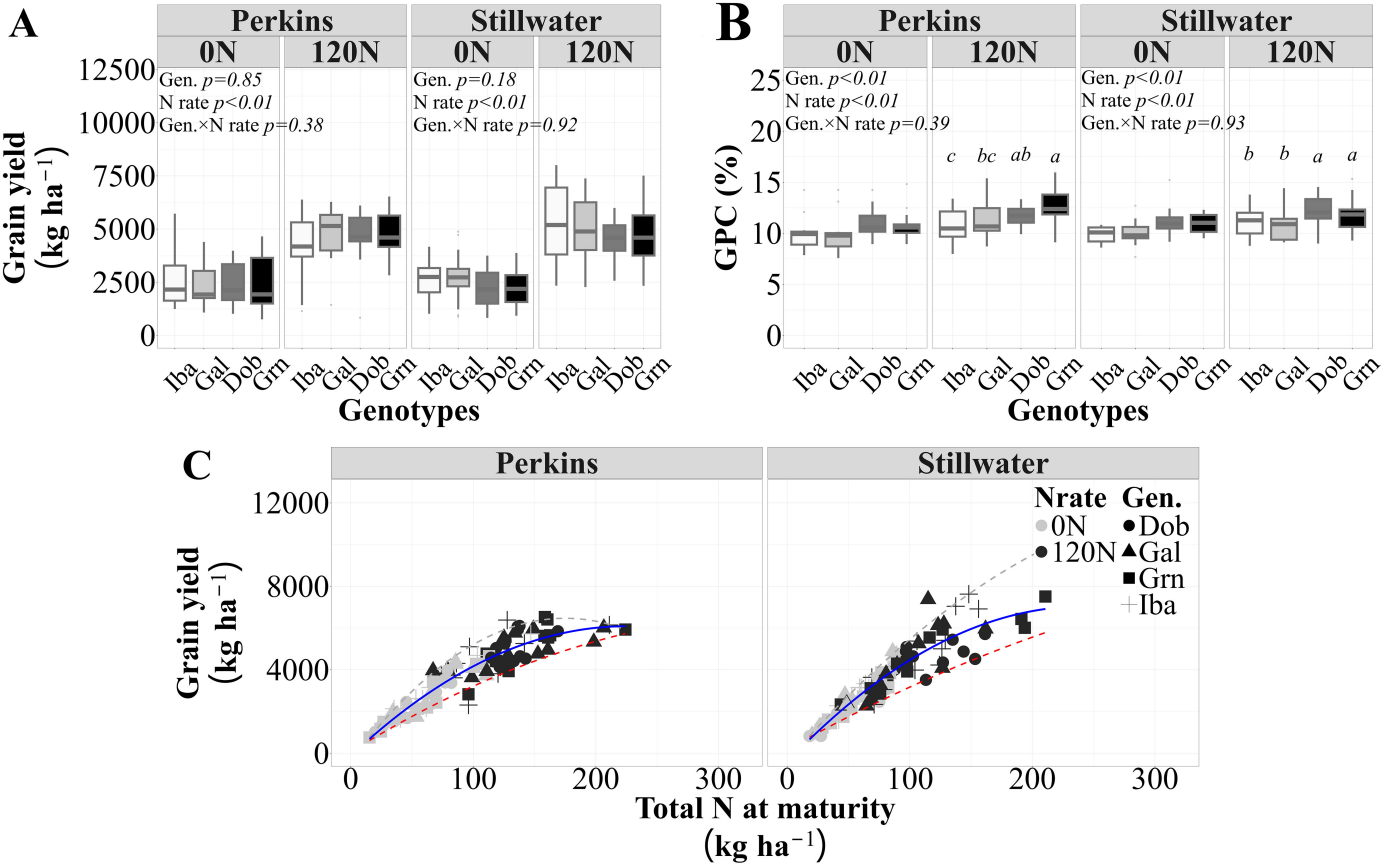
Mean grain yield **(A)**, grain protein concentration **(B)** for each genotype, N rate and site on average of four growing seasons, and the relationship between grain yield and total N accumulation at maturity **(C)** for each site across two N rates (0N and 120N), four genotypes, and four growing seasons (n=96 observations). The solid lines represent the selected models for this relationship (Perkins, y = 0.12x^2^+ 55x-130, R^2^ = 0.83; Stillwater, y = -0.11x^2^ + 62x -418, R^2^=0.84), and the dashed lines are the 5% and 95% quantiles (minimum and maximum N utilization efficiency (i.e., NUtE, grain yield to whole plant N uptake ratio), respectively. The statistical analysis shown in **(B)** indicates that mean values with different letters are statistically different at p<0.05.

The grain yield values ranged from 769 to 8005 kg ha^-1^ with an estimated standard deviation of 1246 kg ha^-1^, and GPC values ranged from 8 to 16% with a standard deviation of 1.5% points across all sources of variation evaluated (i.e., genotypes, N rate, and site-years). As expected, the genotypes tested in this study showed similar yield levels across N rates in both sites (**Figure 1A**) and different GPC at the high N rate in both sites (**Figure 1B**). Iba had the lowest GPC, Gal had a medium GPC, and Dob and Grn with higher GPC values. The high protein genotypes Dob and Grn had greater mean GPCs of 11.4% and 11.6%, respectively, than the low protein genotype Iba, with a mean GPC of 10.3% at the 120N in both sites. They also had greater GPC than the medium protein genotype Gal, with a mean GPC equal 10.6% in Stillwater. A quadratic model better illustrated the relationship between grain yield and total N accumulation at maturity (slope = NUtE) than a linear model (**Supplementary Table S1**). The model showed a quadratic rise towards a maximum yield value, indicating that increases in total N accumulation at maturity resulted in greater yield up to a certain level, after which yield gains diminished as N accumulation continued to rise (**Figure 1C**, **Supplementary Figure S1**). Thus, there was an overall positive and quadratic relationship between grain yield and total N accumulation at maturity, with 87% (for Perkins) and 89% (for Stillwater) of the variation in grain yield due to N rate, genotypes, and growing seasons explained by differences in total N accumulation at maturity (**Figure 1C**). In both sites, the association between grain yield and total N accumulation was investigated to quantify the N Utilization Efficiency (NUtE) of genotypes differing in GPC. This analysis further revealed that increases in yield via increases in NUtE are possible in high grain yield and N accumulation scenarios. Furthermore, in Stillwater, 70% of each high-protein genotypes Dob and Grn data points fell below the trendline, while 54% of the low-protein genotype Iba fell at or above the trendline, specially under low N uptake levels, indicating lower NUtE for high protein genotypes. In Perkins, 54% of each high-protein genotype Grn and Dob datapoints were below the trendline, while 66% of the low-protein genotype Iba were at or above the regression line, indicating a greater NUtE for Iba in comparison to the high-protein genotypes (**Supplementary Figure S2**). Our results suggest that the high NUtE of the low protein genotype Iba may have penalized its GPC, and that the high protein genotypes Dob and Grn seemed to use different pathways to achieve GPC. The higher protein from the genotype Dob did not come from greater N uptake, while it could have for Grn.

### Total N and biomass accumulation in genotypes with different protein levels

**Figure 2:**
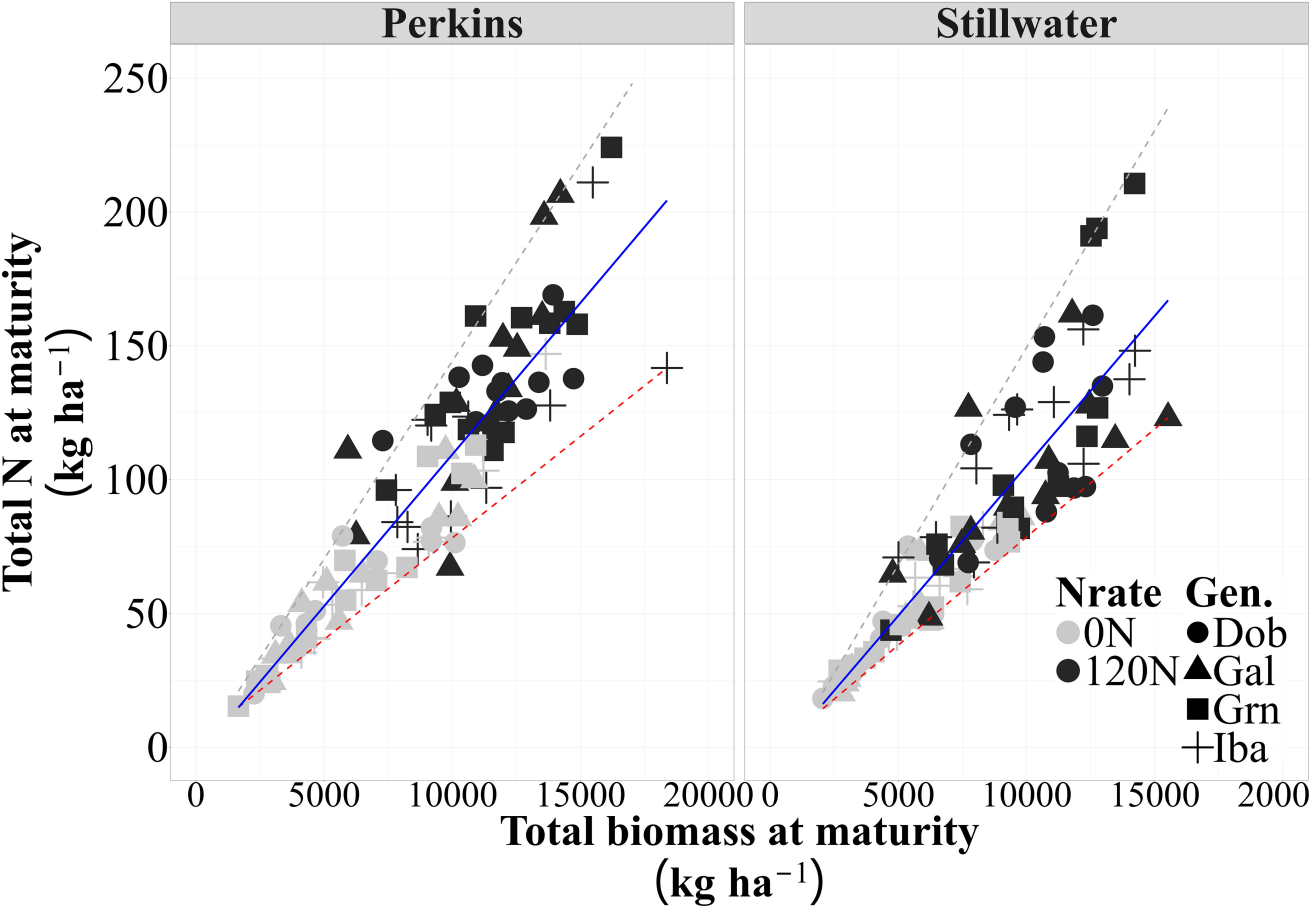
Relationship between total N and biomass accumulation at maturity for each site across four genotypes, two N rates (0N and 120N), and three growing seasons (2020, 2021, 2023) (n=96 observations) (Perkins, y = 0.011x-3.98, R^2^= 0.83, p<0.01 and Stillwater, y = 0.012x-10.9, R^2^=0.77, p<0.01). The solid lines represent the selected models to describe this relationship, and the dashed lines are the 5% and 95% quantiles (minimum and maximum, respectively).

We evaluated whether the high protein genotypes showed increased N and biomass accumulation compared to the low protein genotypes, and whether their accumulation dynamics would change when they were evaluated under low or high N conditions. Nitrogen rate increased biomass and N uptake of all genotypes, but the accumulation patterns between genotypes remained consistent under both low and high N conditions (**Supplementary Tables S4, S5**). A strong positive association was observed between total N accumulation and biomass accumulation at maturity for both sites (**Figure 2**; **Supplementary Figure S3**). A linear model better illustrated the relationship between total N and biomass accumulation at maturity (**Supplementary Table S2**). At both sites, increases in total biomass were related to a corresponding increase in total N accumulation at maturity, irrespective of genotype GPC levels. Based on the r^2^ of this relationship, 83% (Perkins) and 77% (Stillwater) of the variation in N accumulation due to N rate, genotypes, and growing seasons were explained by changes in biomass at physiological maturity. Hence, the stoichiometry of whole plant N uptake and biomass did not change for genotypes with high and low GPC.

### Nitrogen partitioning to the grain

**Figure 3:**
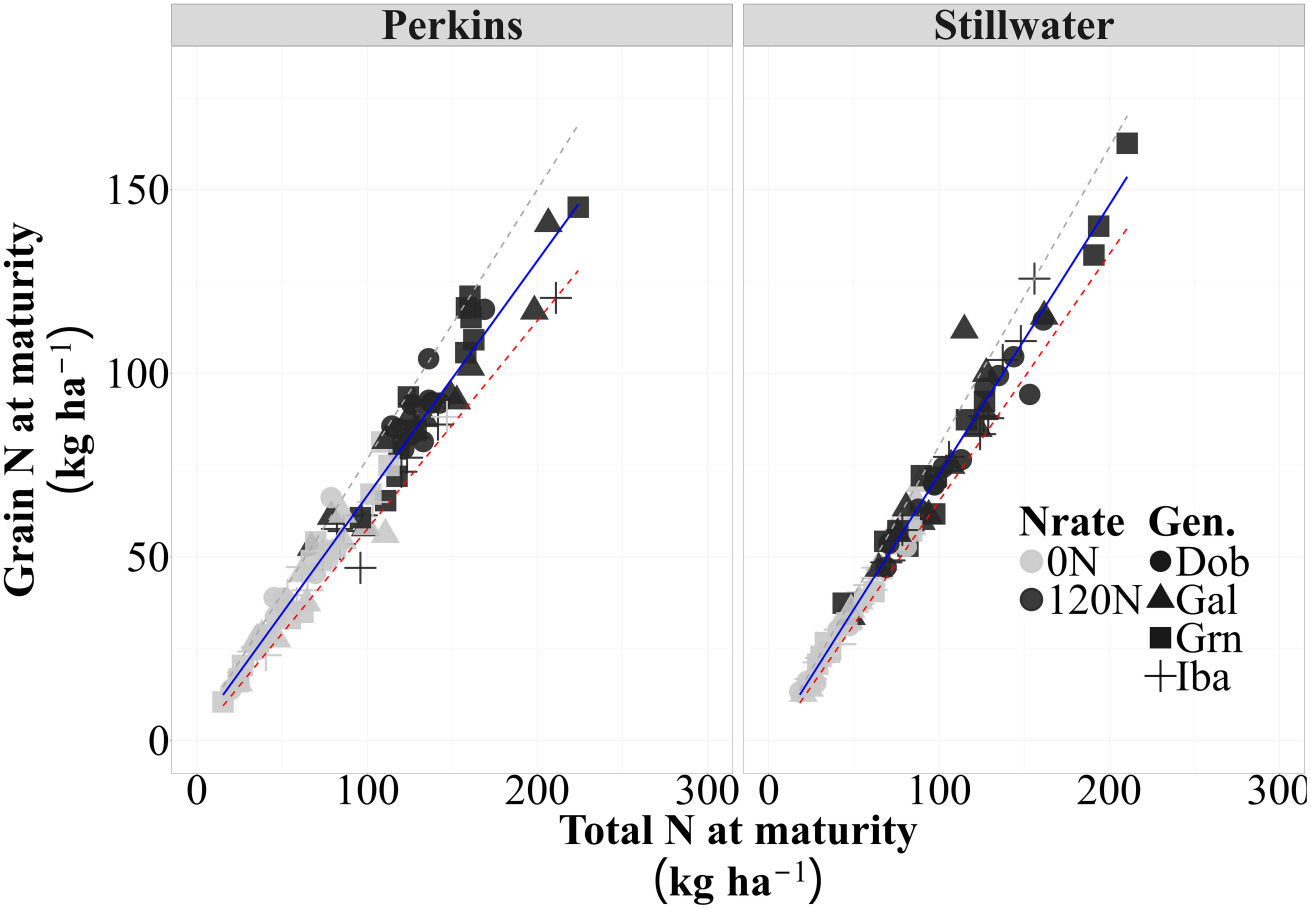
The relationship between grain N and total N accumulation (i.e., slope = N Harvest Index [NHI]) for each site across four genotypes, two N rates (0N and 120N) and three growing seasons (2020, 2021, 2023) (n=96) (Perkins, y = 0.64x+2.6, R^2^ = 0.96; Stillwater, y = 0.77x+3.09, R^2^=0.98). The solid lines are best-fitted functions for this relationship, and the dashed lines are the 5% and 95% quantiles (minimum and maximum, respectively).

There was a strong and positive association between grain N accumulation and total plant N at maturity in both sites (p<0.01), with approximately 96 and 95% of the variation in grain N accumulation across all sources of variation (i.e., N rate, genotypes, and growing seasons) explained by differences in total N accumulation at maturity in both sites (**Figure 3**, **Supplementary Figure S4**). This strong relationship demonstrates that genotypes with higher total N accumulation tended to partition a more significant proportion of their acquired N towards grain and that some genotypic variation may occur. For instance, in Perkins, 50% of the datapoints for the low protein genotype Iba were below the trendline while the 72% of the datapoints for the high protein genotype Dob were at or above the trendline, indicating a greater NHI for Dob as compared to Iba (**Figure 3**; **Supplementary Figure S5**). Based on the slope of this relationship, the average NHI was 63% for Perkins and 67% for Stillwater. The consistency of these strong relationships in both sites (R^2^>0.90 and p<0.05) indicated that the greater NHI observed in the high-protein genotype is relatively stable and less influenced by environmental factors or N availability levels. Therefore, the strong partitioning capability of some high-protein genotypes enables them to maintain high GPC levels while achieving competitive yields. This supports the hypothesis that high-protein genotypes may exhibit increased N partitioning to the grain, irrespective of N availability.

### Grain N sources: Post-flowering N accumulation (PostN), N remobilization to the grain (RemN), remobilized N from individual vegetative organs (RemN_organs_) from anthesis to maturity

**Figure 4:**
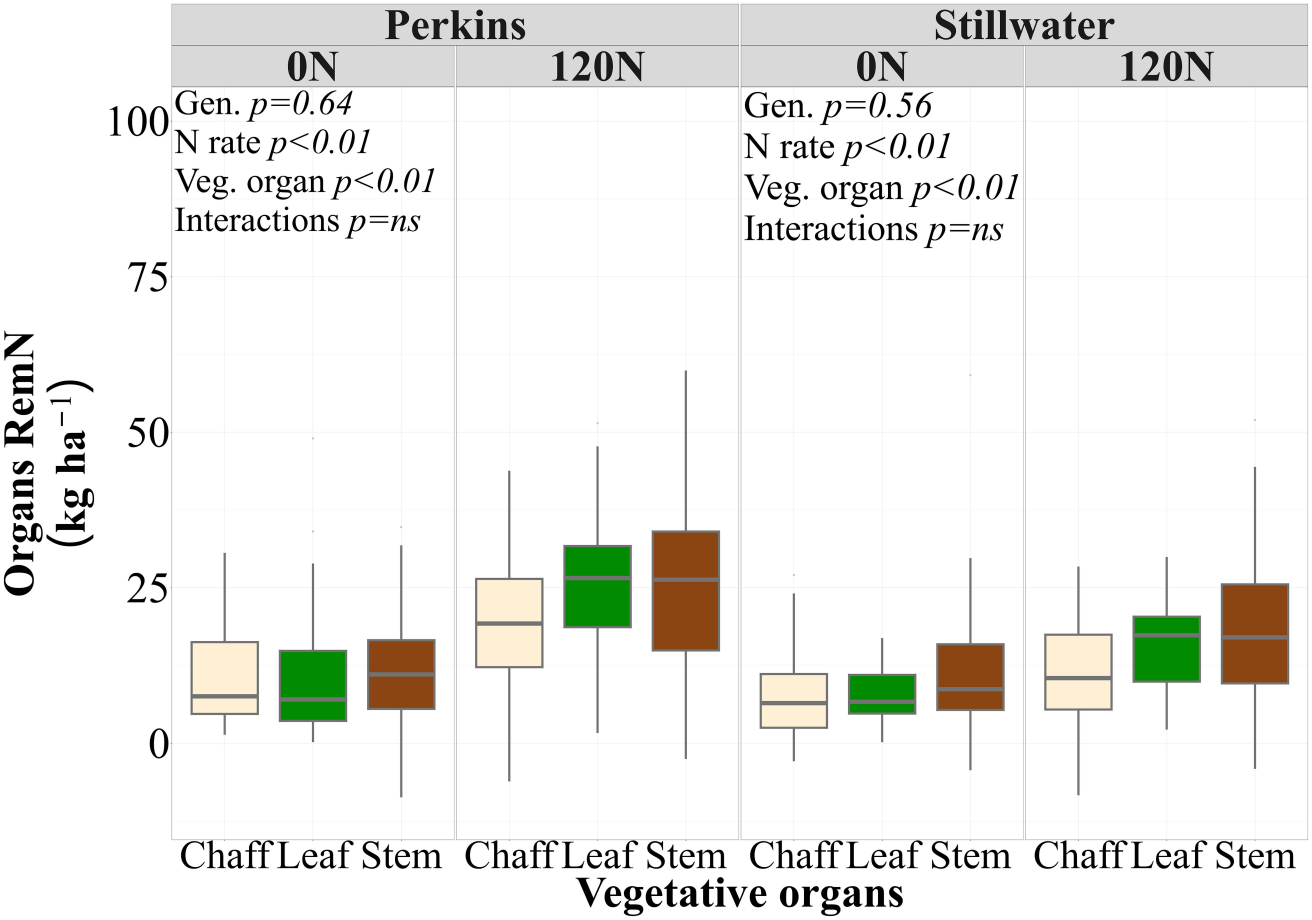
Nitrogen remobilization of each vegetative organ (Organs RemN, kg ha^-1^) at two N rates (0N and 120N) and each site (Perkins and Stillwater) averaged across four genotypes and three growing seasons (2020, 2021, 2023) (n=96). Different letters represent statistical difference among plant organs on average of N rates, genotypes, and growing seasons at *p*< 0.05.

High and low protein genotypes did not change their PostN uptake and N remobilization dynamics when tested under low and high N rate (**Table 3**). While there were no statistical differences between genotypes and their interaction with the N rate, the 120N treatment increased the N remobilized from individual vegetative organs in both sites (p<0.01) (**Table 3**; **Supplementary Table S7**). Thus, genotypes with different GPC levels had similar N remobilization from individual vegetative organs and the sum of all vegetative organs. When comparing the RemN of plant organs averaged over genotypes and growing seasons, the stem remobilized more N from anthesis to maturity than the chaff and was not statistically different from leaf in both sites (**Figure 4**).

**Table 3:** Nitrogen remobilization from vegetative organs from anthesis to maturity (plant component N uptake at anthesis minus plant component N uptake at maturity, RemN_Organs_), the sum of N remobilization from all vegetative organs (Sum RemN_Veg_), whole plant N remobilization (RemN; whole plant N uptake at anthesis minus stover N uptake at maturity) and post-flowering N uptake (PostN, whole plant N uptake at maturity minus whole plant N uptake at anthesis) for each genotype, N rate, and site averaged over four growing seasons.

			RemN_Organs_				Grain N sources	
Site	Genotype	N rate	Stem	Leaf	Chaff	Sum RemN_Veg_	RemN	PostN
			———————— kg ha^-1^ ————————				———— kg ha^-1^———	
**Perkins**	Grn: High Protein		16	13	10	39	72	24
	Dob: High Protein		18	12	10	39	67	21
	Gal: Medium Protein		15	15	14	43	62	26
	Iba: Low Protein		18	15	15	48	64	17
		0N	9b	6b	7b	21b	41b	12b
		120N	25a	21a	17a	64a	91a	32a
	Genotype		ns	ns	ns	ns	ns	ns
	N rate		<0.01	<0.01	<0.01	<0.01	<0.01	<0.01
	Genotype × N rate		ns	ns	ns	ns	ns	ns
**Stillwater**	Grn: High Protein		17	15	8	42	54	32
	Dob: High Protein		14	14	9	38	56	29
	Gal: Medium Protein		17	14	12	47	64	27
	Iba: Low Protein		14	11	10	38	56	29
		0N	13b	9b	11	32b	38b	18b
		120N	19a	18a	14	50a	77a	40a
	Genotype		ns	ns	ns	ns	ns	ns
	N rate		<0.01	<0.01	ns	<0.01	<0.01	<0.01
	Genotype × N rate		ns	ns	ns	ns	ns	ns

Different letters represent the significant statistical differences (HSD) at p< 0.05. ns; no significant statistical differences among treatments (p>0.05).

### Relationship between the proportional contribution of RemN of each organ and NNI

**Figure 5:**
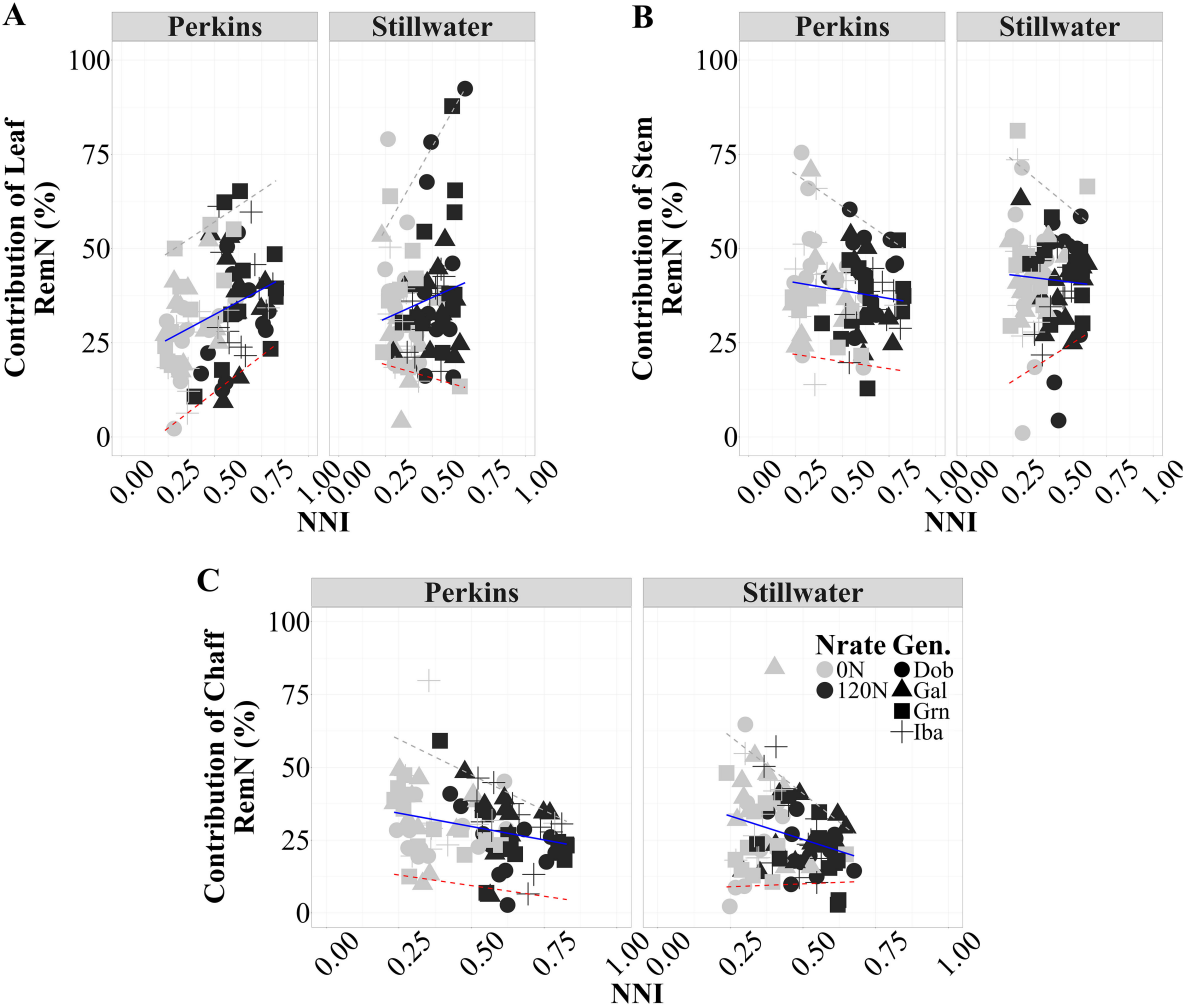
Relationship between contribution RemN_organ_ to Sum RemN_Veg_ and NNI for each site-year across two N rates (0N and 120N) and three growing seasons (2020, 2021, 2023) (n=96). For leaf, Perkins RemN_leaf _vs NNI: 26.5 x + 19.3, R^2^ = 0.12 and Stillwater RemN_leaf _vs NNI: 22.4 x + 25.8, R^2^ = 0.03. For stem, Perkins RemNstem vs NNI: 6.04 x – 44.5, R^2^ < 0.02 and Stillwater RemN_stem_ vs NNI: 6.04 x – 44.5, R^2^ < 0.01. For chaff, Perkins RemN_chaff _vs NNI: 18.4 x – 38.9, R^2^ = 0.07 and Stillwater RemN_chaff_ vs NNI: 32.1 x – 41.3, R^2^ =0.07. The solid lines are best-fitted functions for this relationship, and the dashed lines are the 5% and 95% quantiles (minimum and maximum, respectively).

There was a positive relationship between the proportional contribution of RemN_leaf_ to the Sum RemN_Veg_ (%) and NNI, indicating that under favorable N conditions (higher NNI), a more significant proportion of the Sum RemN_Veg_ was contributed by the leaves relative to other organs (**Figure 5A**; **Supplementary Figure S6**). The high-protein genotypes (Grn and Dob) exhibited a higher proportional contribution of RemN_leaf_ compared to the low-protein genotype (Iba), as evidenced by the 84% of data points above the trend line for high-protein genotypes. This pattern was consistent across both environments, but slightly more evident at the Perkins site, where the high-protein genotype Grn showed greater leaf N uptake at anthesis than the medium-low protein genotypes Gal and Iba (**Supplementary Table S5**). This suggests that genotypes with higher GPC tend to allocate a significant fraction of their Sum RemN_Veg_ from leaves to support N accumulation during grain filling period (*hypothesis 2*). Contrarily, the proportional contribution of RemN_stem_ to Sum RemN_Veg_ exhibited a very weak and negative correlation with NNI (Perkins: r² = 0.02, Stillwater: r² = 0.03) at both sites (**Figure 5B**; **Supplementary Figure S7**). This implies that as N availability improved, the relative contribution of RemN_stem_ decreased, perhaps, due to a shift in N allocation towards other organs, possibly leaf, for optimizing grain protein formation. Notably, the high protein genotypes Grn and Dob exhibited a more pronounced decrease in contribution RemN_stem_ as NNI increased compared to low and medium protein genotypes, as evidenced by their data points above the trend line. This finding suggests potential strategy high protein genotypes employ to prioritize grain N accumulation over vegetative biomass. Similarly, examining the relationship between the contribution of RemN_chaff_ to Sum RemN_Veg_ and NNI, a weak and negative correlation was observed in both Perkins and Stillwater sites (Perkins: r² = 0.13, Stillwater: r² = 0.10) (**Figure 5C**; **Supplementary Figure S8**). This negative association indicates that the chaff contributed a lower proportion of the Sum RemN_Veg_ under more favorable N conditions. Notably, while low and medium protein genotypes (Iba and Gal) showed a more pronounced reduction in the proportional contribution of RemN_chaff_, the high protein genotypes (Grn and Dob) exhibited a distinct pattern. The high-protein genotypes exhibited a distinct pattern in N remobilization under varying NNI conditions. As NNI increased, these genotypes showed a lower proportional contribution of RemN_chaff_ to total remobilized N, with their data points falling below the trend line, especially at higher NNI values. Simultaneously, they demonstrated a higher proportional contribution of RemN_leaves_. This pattern indicates that under improved N status, high-protein genotypes prioritized N remobilization from leaves over chaff, potentially as a strategy to enhance GPC while maintaining efficient overall N use.

## Discussion

### Relationship between grain protein concentration and yield overview

The relationship between GPC and yield in wheat has been extensively studied due to its economic and end-use importance. Multiple studies have been conducted to explore the factors contributing to their negative relationship (Sieling and Kage, 2021; Tanin et al., 2022; Donaire et al., 2023), but further discussion is needed on the N uptake dynamics of genotypes with similar yield levels and different grain protein levels in dryland environments. Generally, the negative relationship between GPC and grain yield arises from competition for energy between C and N compounds in the plant, and N dilution in the grain as grain yield increases (Acreche and Slafer, 2009). The latter occurs when yield gains occur under limited N conditions (Munier‐Jolain and Salon, 2005). As demonstrated in our research and previous studies, genotypes differ in their magnitude of trade-off between GPC and yield (Cox et al., 1986; Bogard et al., 2010). Thus, understanding the physiological traits that enable genotypes to maintain or improve yield with minimal impact on GPC is relevant for the hard red wheat industry.

### Genotypes performance under low and high N rates

Wheat genotypes can perform differently under low and high N conditions. Cormier et al. (2016) found that certain wheat genotypes exhibited better tolerance to N deficiency, maintaining higher biomass and grain yield under low N conditions. However, in our study, genotypes did not differ in any of the traits evaluated under low N. This discrepancy between our findings could be attributed to several factors, including the severity of N stress in our experiment, which may have been more extreme than in other studies, potentially masking genetic variation. Gaju et al. (2014) observed that under severe N stress, relationships between N uptake, remobilization, and yield components changed compared to conditions of mild N stress or high N availability. Furthermore, genotype and environment interactions play a crucial role in the expression of N use efficiency (NUE) traits. Teng et al. (2022) reported that genetic factors accounted for a more significant portion of the variation in GPC in wheat. Gaju et al. (2011, 2014) observed that wheat genotypes differed in their ability to maintain GPC under varying N supply, with some genotypes maintaining higher grain N concentrations through more efficient N remobilization from vegetative tissues. Our study corroborates with previous findings regarding varied genotypic responses under high N conditions, particularly regarding yield and GPC. This is consistent with Bogard et al. (2010), who reported that high N conditions allowed for continued N uptake during grain filling, resulting in increased grain protein accumulation for some genotypes. Barraclough et al. (2010) found that high-yielding wheat genotypes often had lower GPC, particularly under high N supply. Regarding the differences in grain N sources under low and high N rates, Kichey et al. (2007) observed that under high N conditions, some genotypes showed increased post-anthesis N uptake, which contributed more significantly to grain N accumulation compared to N remobilization from vegetative tissues. This observation does not align with our results, where N remobilization increased as compared to post-anthesis N uptake under high N conditions.

The greater NUtE exhibited by low protein genotype Iba aligns with the concept of a physiological tradeoff between NUtE and GPC which is similar to findings of previous studies (Ivić et al., 2021; Teng et al., 2022; Thompson et al., 2022). Future research efforts should focus on identifying physiological traits and management strategies to optimize both NUtE and GPC to maintain or increase yields, reduce environmental nitrogen loss, and achieve desirable protein levels while considering economic factors.

### Total biomass and N accumulation at physiological maturity

Variations in GPC among genotypes may be driven by differences in other traits or N partitioning rather than total biomass. Dier et al. (2022) suggested that greater GPC in some genotypes may originate from their ability to partition N in the grain more efficiently. The subtle difference observed in Grn, however, suggests that some high-protein genotypes may indeed utilize increased N uptake as a strategy to enhance GPC. This finding underscores the complexity of the mechanisms underlying GPC variations and highlights the importance of considering both general trends and trait-specific strategies when breeding for improved grain quality.

### Nitrogen partitioning to the grain

Plants with greater total N accumulation at maturity tend to allocate more N to grain, as consistently observed in wheat across various studies (Barraclough et al., 2010; De Oliveira Silva et al., 2020). The relationship between grain N uptake and total N uptake, as reflected in the NHI observed in our study, aligns with and extends previous research findings. Consistent with studies by Montemurro et al. (2006) and Rahimizadeh et al. (2010), we found that NHI plays a critical role in influencing GPC across diverse wheat genotypes and environments. Our results revealed that high-protein genotypes (Grn and Dob) demonstrated higher NHI compared to the low-protein genotype (Iba), supporting our hypothesis that high-protein genotypes exhibit increased N partitioning to the grain relative to low-protein genotypes. A higher NHI indicates that more of the absorbed N is being utilized for grain protein production, potentially reducing the amount of excess nitrogen left in crop residues or lost to the environment (Kirika, 2021). This efficient use of N can contribute to reducing environmental nitrogen pollution, a major concern in agricultural systems.

### Grain N pathways: post-flowering N accumulation and N remobilization to the grain

While numerous studies have investigated management strategies and traits that could help in enhancing both GPC and grain yield without substituting one for the other (Dhillon et al., 2020; Lollato et al., 2021; Kartseva et al., 2023), no one has investigated the different remobilization strategies of genotypes with high and low protein under zero and high N conditions nor have they detailed the organ-specific contributions to N remobilization in modern wheat varieties. Across all genotypes, RemN_leaf_ and RemN_stem_ emerged as the primary contributors to grain N, followed by RemN_chaff_. This aligns with previous research showing stems and leaves as significant sources of RemN during grain-filling (Masoni et al., 2007; Egle et al., 2015). Interestingly, the effect of the N rate on RemN_chaff_ varied depending on the environment, suggesting that environmental or management factors may influence its contribution (Dordas, 2009). Thus, the lack of significant differences among genotypes for PostN and RemN traits suggests they responded similarly to environmental conditions, underscoring the importance of N management in optimizing grain yield and GPC. These findings indicate that N availability, rather than genotypic differences, is the primary driver of these processes across all genotypes studied.

### Proportional contribution organ RemN to total RemN and their relationship with NNI

Consistently with previous research (Kichey et al., 2007), leaf N remobilization plays a critical role during grain filling to sustain plant N status and attain protein levels. Similar findings from studies by Barraclough et al. (2010) and Gaju et al. (2011, 2014) also posited that in wheat, remobilizing N from the leaves during grain filling determines the ultimate grain N concentration. Conversely, the negative correlation between RemN_stem_ contribution and NNI at both sites, suggesting a shift in nitrogen allocation as availability increases, consistent with findings by Barraclough et al. (2010). High-protein genotypes exhibited a more pronounced decrease in RemN_stem_ contribution with increasing NNI, suggesting a distinct physiological strategy that may favor grain protein accumulation over vegetative growth (Triboi and Triboi-Blondel, 2002). Similarly, RemN_chaff_ decreased as NNI increased, with high-protein genotypes showing lower RemN_chaff_ but higher NNI values. These results support our hypothesis that N remobilization patterns differ between high and low protein genotypes, aligning with Pask et al. (2012) and Gaju et al. (2014) proposition that high-GPC genotypes may have evolved efficient N remobilization mechanisms.

The enhanced leaf N remobilization observed in high-protein genotypes under increased NNI is particularly noteworthy, corroborating the importance of leaf senescence and N remobilization in determining final GPC (Masclaux-Daubresse et al., 2010). These differential N partitioning strategies, especially the prioritization of leaf N remobilization in high-protein genotypes, likely contribute to their ability to maintain elevated GPC while responding to N availability conditions (Cormier et al., 2016). These findings have important implications for breeding programs aimed at developing wheat varieties with improved nitrogen use efficiency and high GPC. However, future research should consider how these N partitioning strategies may be modulated under different environmental stresses, particularly in the context of climate change and increasing water scarcity in wheat-growing regions.

## Conclusion

Our study addresses the challenge of understanding nitrogen dynamics in wheat by investigating whether modern high-protein genotypes exhibited increased N accumulation (also expressed as N nutrition index, NNI) and partitioning (including remobilization from vegetative organs) compared to low-protein genotypes under low and high N conditions.

Our results support the hypothesis that N remobilization patterns differ between high- and low-protein genotypes. Leaf N remobilization played a critical role during grain filling, sustaining plant N status and contributing to protein levels. High-protein genotypes demonstrated a prioritization of N remobilization from leaves as N conditions improved, likely contributing to their ability to maintain elevated GPC. Specifically, Grn tended to achieve high protein by increasing total N uptake, while Dob exhibited a trend toward higher N partitioning to grain (NHI). These differential N partitioning strategies support the hypothesis that high-protein genotypes exhibit increased N partitioning to the grain compared to low-protein genotypes. The greater N utilization efficiency (NUtE) observed in the genotype Iba likely contributed to its lower GPC, emphasizing the trade-off between NUtE and GPC. Future research should further elucidate the genetic and physiological mechanisms underlying these N partitioning strategies in different organs during the entire growing season in high-protein genotypes. This knowledge could lead to more targeted breeding efforts and management practices that balance the competing demands of high yield, high GPC, and efficient N use in wheat production.

## Data Availability

The original contributions presented in the study are included in the article/**Supplementary Material**. Further inquiries can be directed to the corresponding author.
