# Supplementary material for: Nitrogen uptake dynamics of high and low protein wheat genotypes
Source: Front Plant Sci. 2024 Dec 16;15:1493901. doi: 10.3389/fpls.2024.1493901 (PMC11686436; doi:10.3389/fpls.2024.1493901)
Supplement: Supplementary file 1 [file DataSheet1.pdf]

Table 1. Statistical comparisons between quadratic and linear models for the relationship between grain yield and whole plant N uptake at maturity

	Perkins					Stillwater				
**Model**	**R^2**	**AIC**	**BIC**	**Std. error of estimate**	**MS Error**	**R^2**	**AIC**	**BIC**	**Std. error of estimate**	**MS Error**
Quadratic	0.87	1491	1501	222	308659	0.89	1492	1503	209	313690
Linear	0.82	1519	1527	148	417374	0.85	1519	1527	139	419712

Table 2. Statistical comparisons between quadratic and linear models for the relationship between whole plant N uptake at maturity and whole plant biomass at maturity

	Perkins					Stillwater				
**Model**	**R^2**	**AIC**	**BIC**	**Std. error of estimate**	**MS Error**	**R^2**	**AIC**	**BIC**	**Std. error of estimate**	**MS Error**
**Quadratic**	0.83	850	860	10	388	0.77	868	879	12	472
**Linear**	0.83	848	855	5	383	0.76	867	875	6	472

Table 3. Statistical comparisons between quadratic and linear models for the relationship between Grain N uptake at maturity and Whole plant N uptake at maturity

	Perkins					Stillwater				
**Model**	**R^2**	**AIC**	**BIC**	**Std. error of estimate**	**MS Error**	**R^2**	**AIC**	**BIC**	**Std. error of estimate**	**MS Error**
**Quadratic**	0.96	633	643	2.5	40	0.96	633	643	2.4	41
**Linear**	0.96	632	639	1.4	40	0.95	658	665	1.9	53

Table 4. Aboveground biomass (kg ha^{-1}) for individual plant organs (stem, leaf, spike, chaff) and whole plant at anthesis and physiological maturity stages for each genotype, N rate and site on average of four growing seasons.

Environment	Genotypes	N rates	Anthesis				Maturity				
			Stem	Leaf	Spike	Total	Stem	Leaf	Chaff	Grain	Total
			----------------- kg ha^{-1}------------------				----------------- kg ha^{-1}------------------				
Perkins	Grn: High Protein		3944	1514	1085	6701	3659AB	1099	1381	3140	9342
	Dob: High Protein		4206	1264	1053	6533	3892A	1100	1336	3084	8628
	Gal: Medium Protein		3842	1245	1295	6574	2914B	946	1364	3128	9342
	Iba: Low Protein		3869	1240	1225	6538	3143AB	836	1167	2931	3389
		0N	2862b	884b	855b	4612b	2438b	676	945b	2133b	5948b
		120N	5118a	1762a	1483a	8623a	4366a	1315a	1679a	4088a	11651a
	Genotype		ns	ns	ns	ns	<0.01	ns	ns	ns	ns
	N rate		<0.01	<0.01	<0.01	<0.01	<0.01	<0.01	<0.01	<0.01	<0.01
	Genotype × N rate		ns	ns	ns	ns	ns	ns	ns	ns	ns
Stillwater	Grn: High Protein		3153	869	926B	4948	2486	852	1389	2989	8106
	Dob: High Protein		3459	916	1016AB	4948	2449	779	1446	2922	7799
	Gal: Medium Protein		3292	967	1274A	5532	2151	842	1722	3343	7633
	Iba: Low Protein		3296	908	1122AB	5326	2229	814	1635	3417	7899
		0N	2642b	696b	844b	4181 b	1739b	588b	1042b	2135b	5869b
		120N	3958a	1134a	1326a	6417a	2919a	1056a	2054a	4201a	9849a
	Genotype		ns	ns	<0.05	ns	ns	ns	ns	ns	ns
	N rate		<0.01	<0.01	<0.01	<0.01	<0.01	<0.01	<0.01	<0.01	<0.01
	Genotype × N rate		ns	ns	ns	ns	ns	ns	ns	ns	ns

Different letters represent the significant difference (Tukey, HSD) at the 0.05 probability level. Capital case letters are used for genotype differences and lowercase for N rates.
ns: nonsignificant at the 0.05 probability level

Table 5. Aboveground N uptake (kg ha^{-1}) for individual plant organs (stem, leaf, spike, chaff) and whole plant at anthesis stage on average of four growing seasons and physiological maturity stage for each genotype, N rate, and site on average of three growing seasons.

Environment	Genotypes	N rates	Anthesis				Maturity				
			Stem	Leaf	Spike	Total	Stem	Leaf	Chaff	Grain	Total
			---------------- kg ha^{-1}-----------------				---------------- kg ha^{-1}-----------------				
Perkins	Grn: High Protein		31	30A	22	82	13	8	11	66A	101
	Dob: High Protein		33	22B	24	76	13	8	10	61AB	94
	Gal: Medium Protein		27	22B	26	74	15	9	12	61AB	98
	Iba: Low Protein		30	24B	21	78	15	7	10	53B	90
		0N	18b	13b	15b	47b	10b	4b	7b	38b	60b
		120N	42a	36a	31a	109a	18a	12a	15a	82a	131a
	Genotype		ns	<0.05	ns	ns	<0.01	ns	ns	0.1	ns
	N rate		<0.01	<0.01	<0.01	<0.01	<0.01	<0.01	<0.01	<0.01	<0.01
	Genotype × N rate		ns	ns	ns	ns	ns	ns	ns	0.15	ns
Stillwater	Grn: High Protein		24	17	15B	57	10	5	8	60	84
	Dob: High Protein		26	17	17AB	59	11	5	8	59	82
	Gal: Medium Protein		22	16	20A	59	9	5	8	61	84
	Iba: Low Protein		22	15	17AB	55	10	5	8	62	81
		0N	16b	10b	13b	39b	6b	3b	6b	39b	53b
		120N	76a	23a	22a	76a	13a	7a	11a	83a	113a
	Genotype		ns	ns	<0.05	ns	ns	ns	ns	ns	ns
	N rate		<0.01	<0.01	<0.01	<0.01	<0.01	<0.01	<0.01	<0.01	<0.01
	Genotype × N rate		ns	ns	ns	ns	ns	ns	ns	ns	ns

Different letters represent the significant difference (Tukey, HSD) at the 0.05 probability level. Capital case letters are used for genotype differences and lowercase for N rates.
ns: nonsignificant at the 0.05 probability level

Table 6. Aboveground N concentration (Nc, %) for individual plant organs (stem, leaf, spike, chaff) and whole plant at anthesis stage on average of four growing seasons and physiological maturity stage for each genotype, N rate, and site on average of three growing seasons.

			Anthesis				Maturity				
**Environment**	**Genotypes**	**N rates**	**Stem**	**Leaf**	**Spike**	**Total**	**Stem**	**Leaf**	**Chaff**	**Grain**	**Total**
			%				%				
	Grn: High Protein		0.7	1.9A	1.7A	4.4A	0.4B	0.83	0.8	2.0A	4.0
	Dob: High Protein		0.8	1.7AB	1.7A	4.2AB	0.4B	0.77	0.7	2.0A	3.8
	Gal: Medium Protein		0.7	1.7B	1.6B	4.0B	0.5A	0.85	0.8	1.9B	3.9
	Iba: Low Protein		0.8	1.7AB	1.6B	4.2AB	0.42AB	0.85	0.8	1.8B	3.8
**Perkins**		0N	0.6b	1.5b	1.6b	3.7b	0.3b	0.7b	0.67b	1.8b	3.56b
		120N	0.8a	2.0a	1.8a	4.7a	0.4a	0.9a	0.8a	2.1a	4.2a
	Genotype		ns	<0.05	0.07	0.07	<0.01	ns	ns	0.09	ns
	N rate		<0.01	<0.01	<0.01	<0.01	<0.01	<0.01	<0.01	<0.01	<0.01
	Genotype × N rate		ns	ns	ns	ns	ns	ns	ns	0.15	ns
	Grn: High Protein		0.7	1.9A	1.6AB	4.3A	0.34	0.6	0.7	2A	3.7
	Dob: High Protein		0.7	1.8AB	1.6A	4.2AB	0.4	0.7	0.7	2A	3.8
	Gal: Medium Protein		0.7	1.6B	1.6AB	3.9B	0.4	0.7	0.6	1.8B	3.5
**Stillwater**	Iba: Low Protein		0.6	1.7AB	1.5B	3.9B	0.5	0.7	0.6	1.8B	3.7
		0N	0.6b	1.5b	1.5b	3.6b	0.4b	0.6b	0.7	1.8b	3.4b
		120N	0.8a	2.1a	1.7a	4.5a	0.5a	0.8a	0.8	2.0a	3.9a
	Genotype		ns	<0.05	<0.05	<0.05	ns	ns	ns	<0.01	ns
	N rate		<0.01	<0.01	<0.01	<0.01	<0.05	<0.01	ns	<0.01	<0.01
	Genotype × N rate		ns	ns	ns	ns	ns	ns	ns	ns	ns

Different letters represent the significant difference (Tukey, HSD) at 0.1 probability level. Capital case letters are used for genotype differences and lowercase for N rates.

ns: nonsignificant at 0.1 probability level

Table 7. Remobilized N from each plant organ for each genotype, N rate, and site on average of three growing seasons.

			**Perkins**	**Stillwater**
Genotypes	N rates	Plant organs	RemN	RemN
			------------- kg ha^{-1}	--------------
Iba			23	14
Gal			19	16
Dob			21	15
Grn			21	15
	0		13 b	11b
	120		25 b	21a
		Chaff	18 B	12B
		Leaf	20 AB	14AB
		Stem	22 A	17A
Gen.			ns	ns
**N rates**			**<0.01**	**<0.01**
**Plant organs**			**<0.01**	**<0.01**
Gen. × N rate			ns	ns
Gen. × Plant organ			ns	ns
N rate × Plant organ			ns	ns
Gen. × N rate × Plant organ			ns	ns

Different letters represent significant statistical difference (Tukey, HSD) at the 0.05 probability level. Capital case letters used for plant organs differences, and lowercase for N rates.
ns: nonsignificant at the 0.05 probability levels

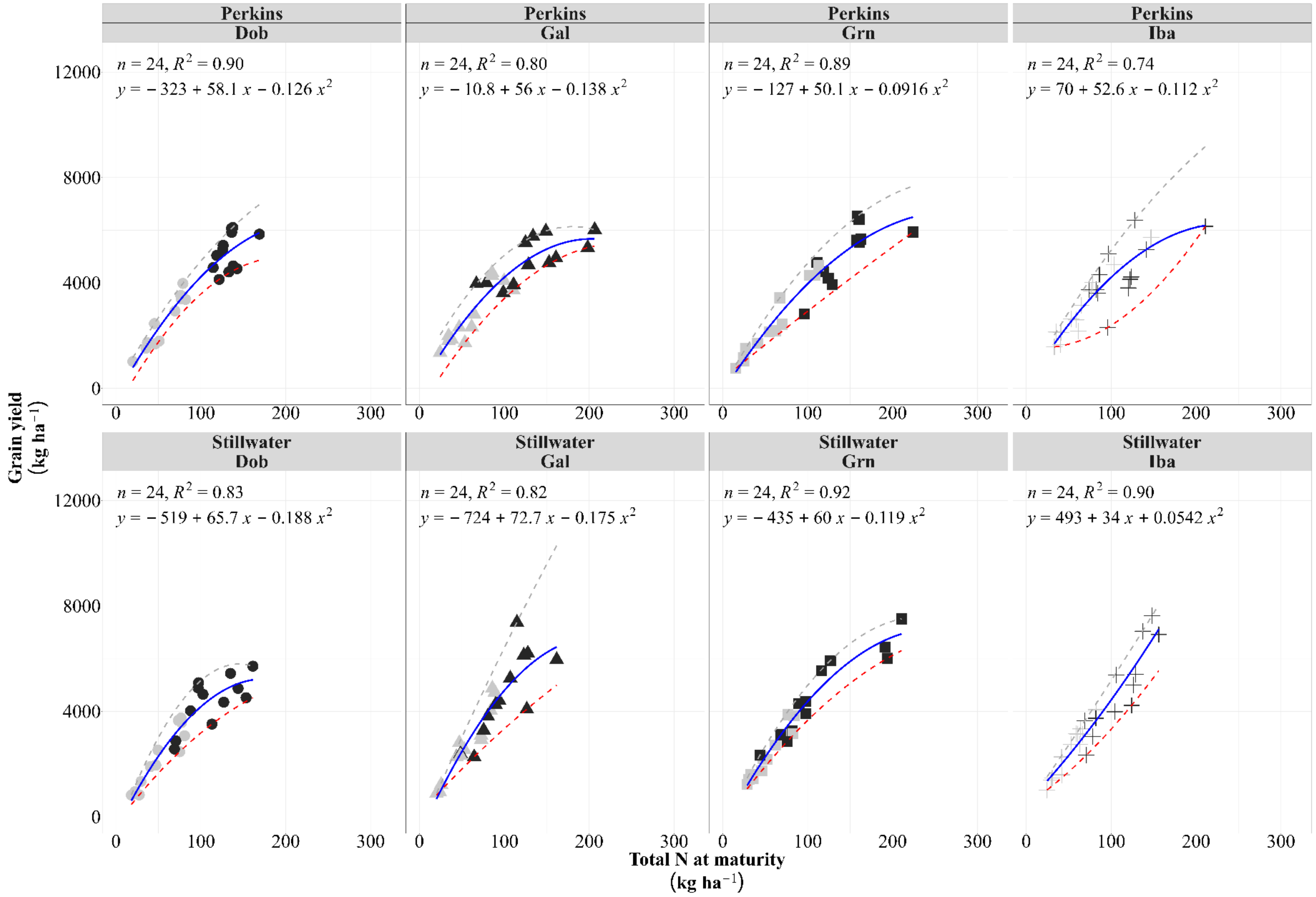

Figure 1. The relationship between grain yield and total N accumulation at maturity for each genotype and site across two N rates (0N and 120N) and three growing seasons (n=24 observations).

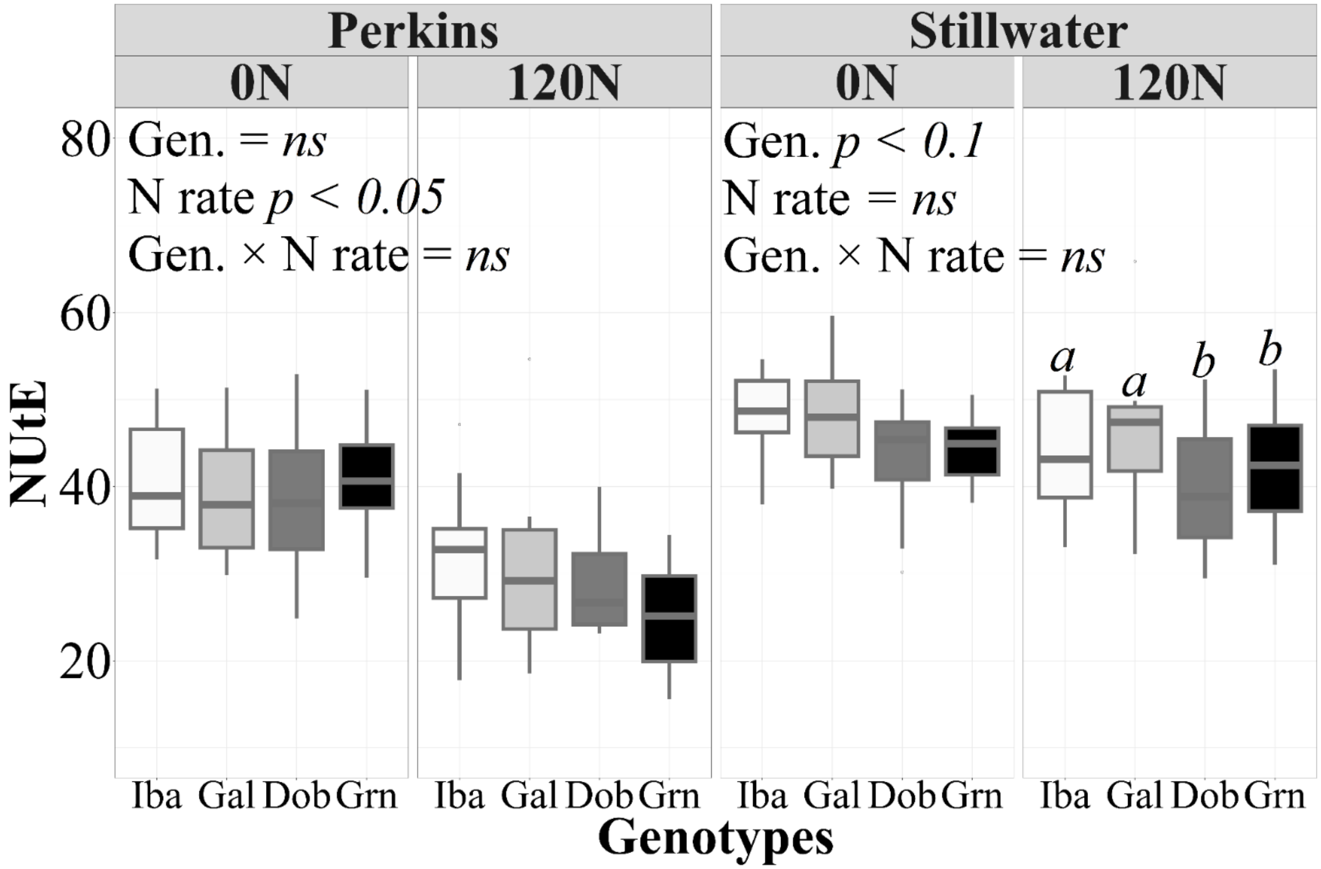

Figure 2. Mean Nitrogen Utilization Efficiency (NUtE, kg kg^{-1}) of each genotype, N rate, and site averaged across three growing seasons. Different letters represent statistical difference among genotypes on average of N rates and three growing seasons at p< 0.05.

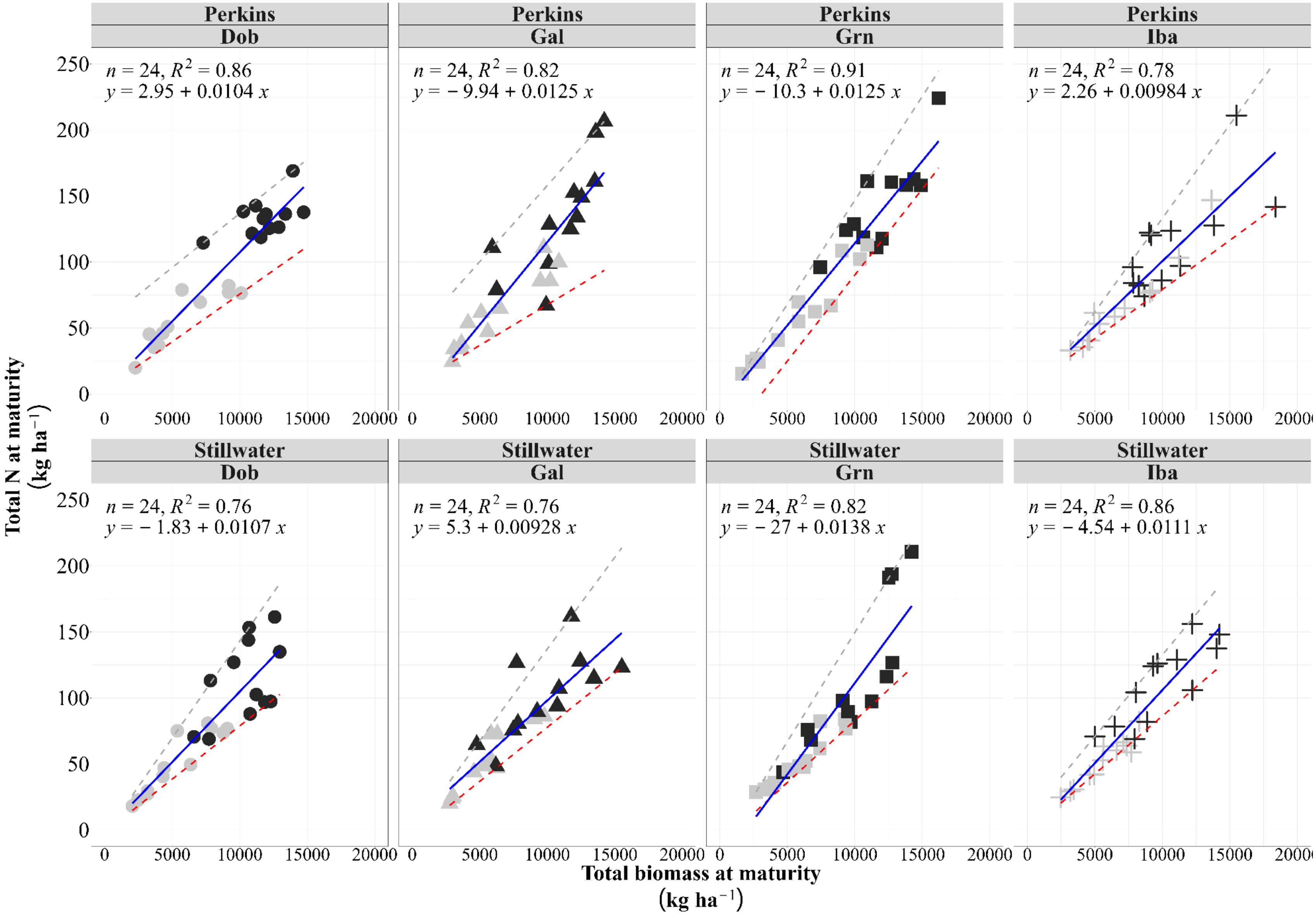

Figure 3. The relationship between total N accumulation and total biomass at maturity for each genotype and site across two N rates (0N and 120N) and three growing seasons (n=24 observations).

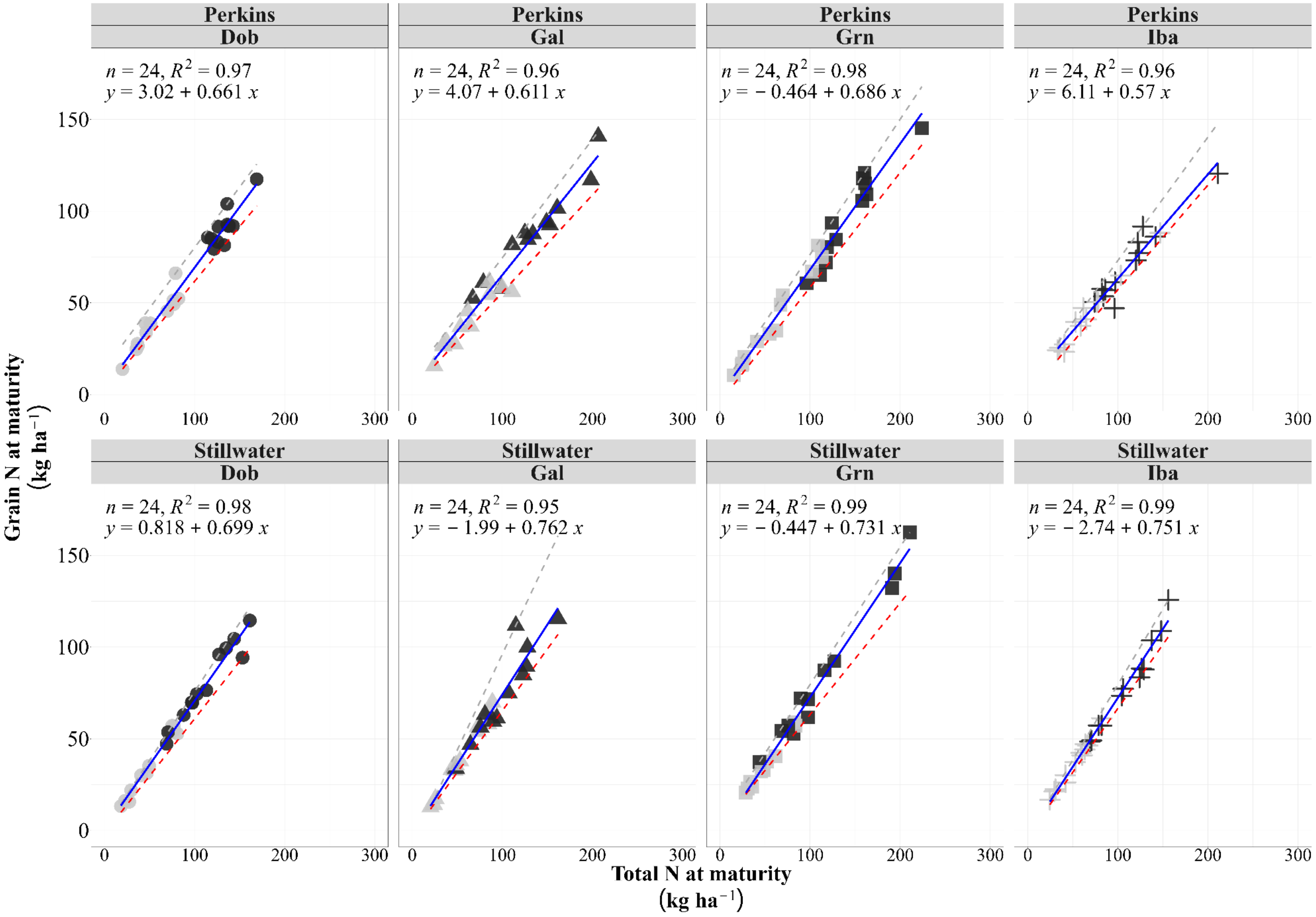

Figure 4. The relationship between grain N and total N accumulation at maturity for each genotype and site across two N rates (0N and 120N) and three growing seasons (n=24 observations).

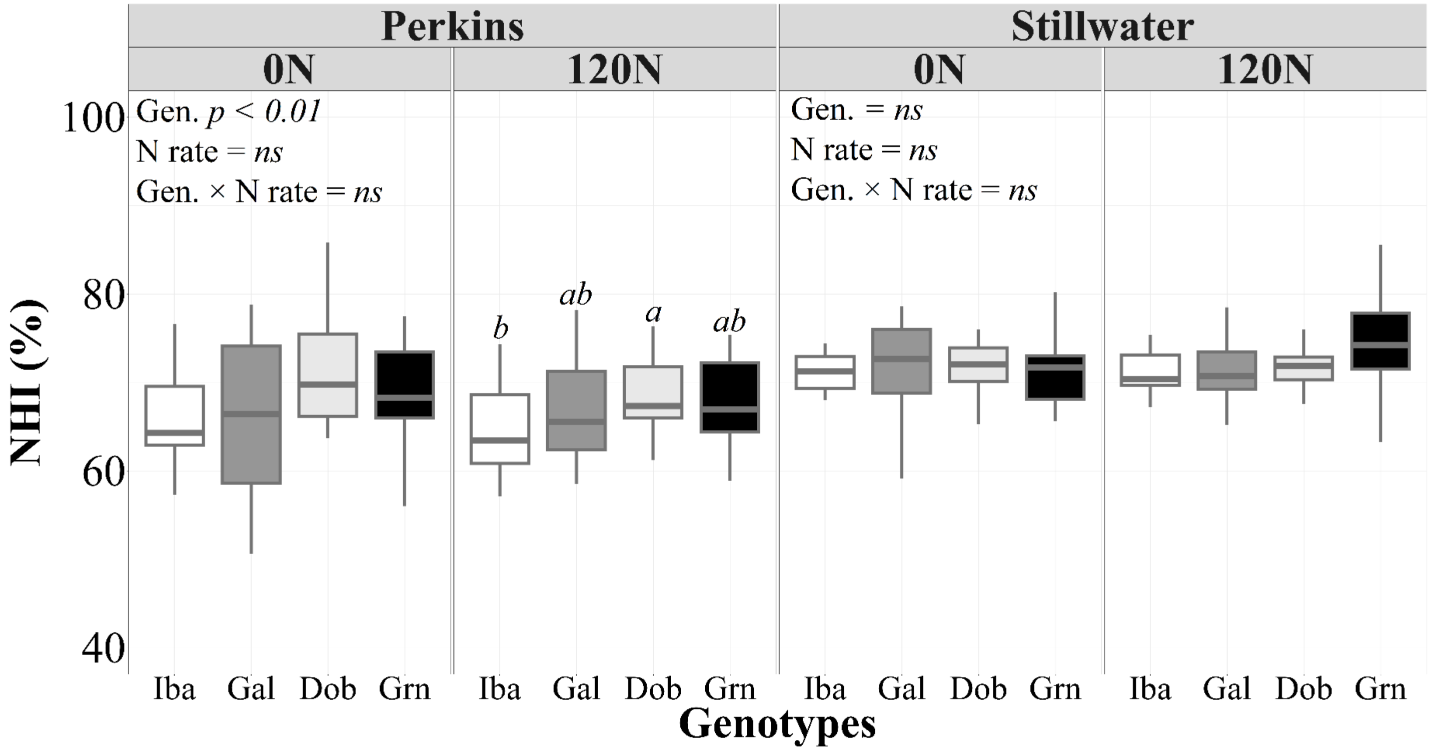

Figure 5. Nitrogen Harvest Index (NHI, %) for each genotype, N rate, and site across three growing seasons. Different letters represent statistical difference among four genotypes on average of N rates and growing seasons (p< 0.05).

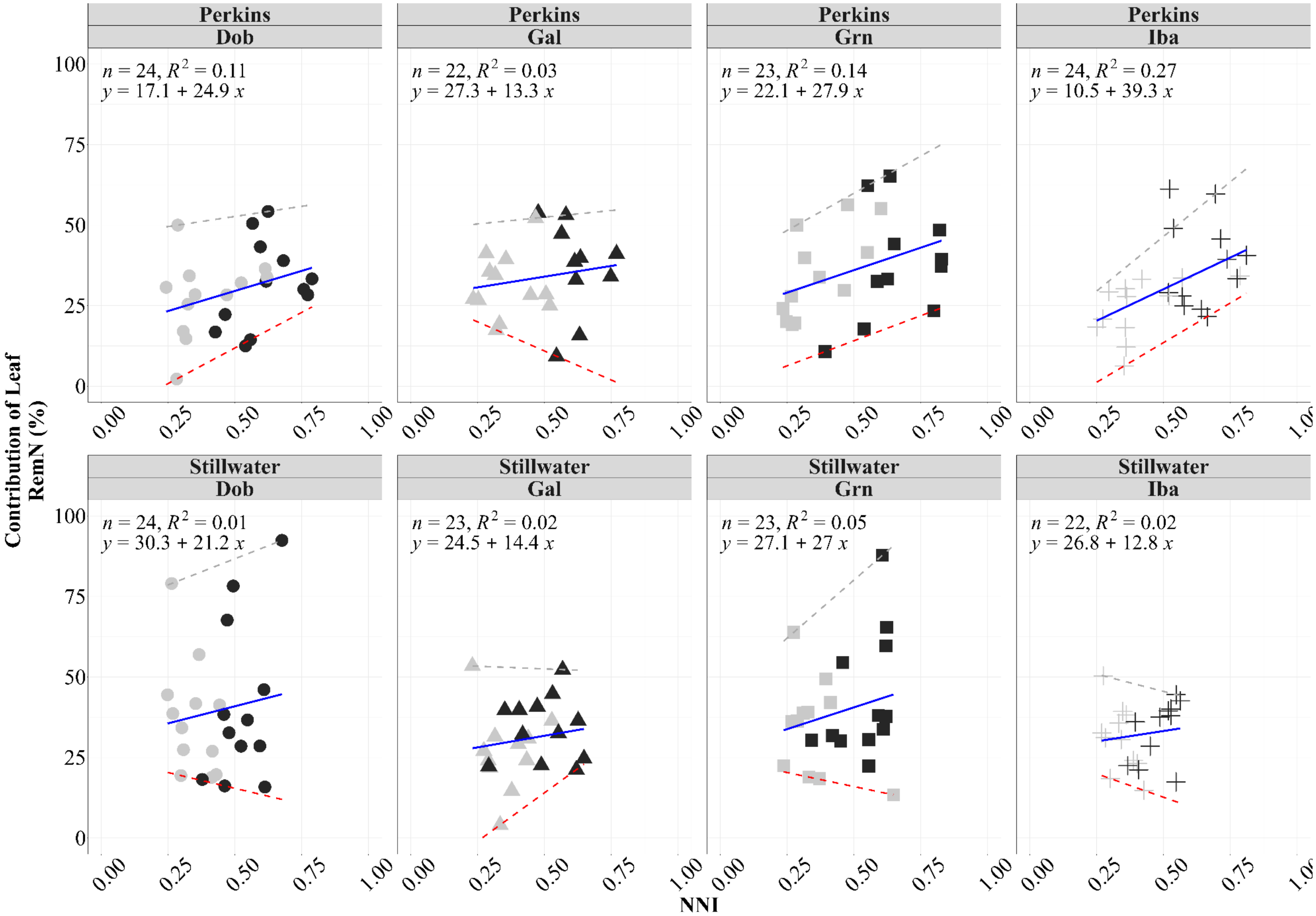

Figure 6. The relationship between the contribution of leaf RemN and NNI for each genotype and site across two N rates (0N and 120N) and three growing seasons (n=24 observations).

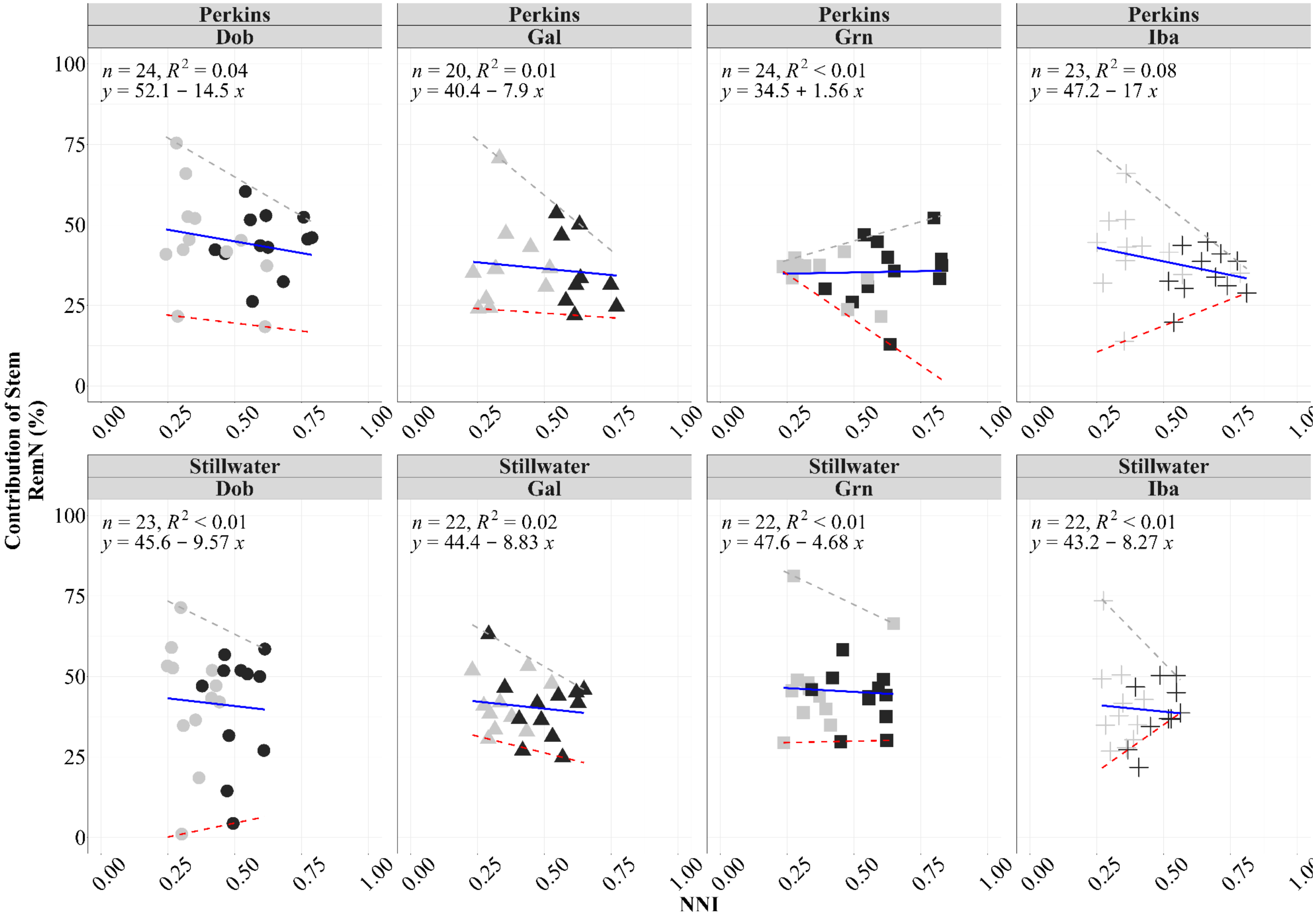

Figure 7. The relationship between the contribution of stem RemN and NNI for each genotype and site across two N rates (0N and 120N) and three growing seasons (n=24 observations).

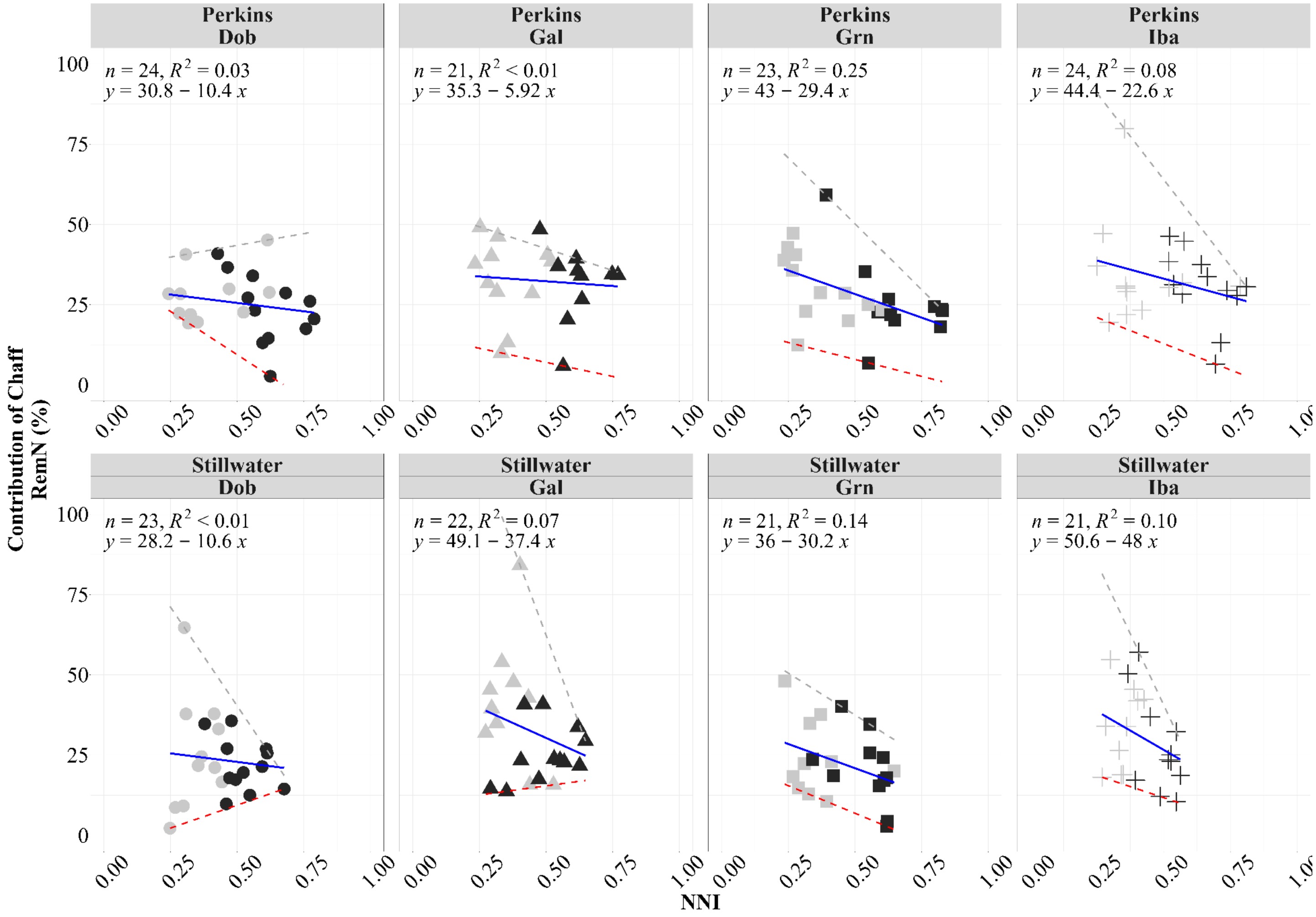

Figure 8. The relationship between the contribution of chaff RemN and NNI for each genotype and site across two N rates (0N and 120N) and three growing seasons (n=24 observations).
